# Optimizing team science in an academic medical center: A qualitative examination of investigator perspectives

**DOI:** 10.1017/cts.2023.3

**Published:** 2023-01-23

**Authors:** Hilary L. Surratt, Janet K. Otachi, Emily Slade, Philip A. Kern, Victoria King, Thomas H. Kelly, Robert S. DiPaola

**Affiliations:** 1 Department of Behavioral Science, University of Kentucky College of Medicine, Lexington, KY, USA; 2 Center for Clinical and Translational Science, University of Kentucky, Lexington, KY, USA; 3 University of Kentucky Health Care, Psychiatric Services, Lexington, KY, USA; 4 Department of Biostatistics, University of Kentucky, College of Public Health, Lexington, KY, USA; 5 Department of Internal Medicine, Division of Endocrinology, University of Kentucky College of Medicine, Lexington, KY, USA; 6 Department of Internal Medicine, Division of Cardiology, University of Kentucky College of Medicine, Lexington, KY, USA; 7 Office of the Provost, University of Kentucky, Lexington, KY, USA

**Keywords:** Team science, career development, program evaluation, pilot programs, physician scientist

## Abstract

**Introduction:**

Optimizing the effectiveness of a team-based approach to unite multiple disciplines in advancing specific translational areas of research is foundational to improving clinical practice. The current study was undertaken to examine investigators’ experiences of participation in transdisciplinary team science initiatives, with a focus on challenges and recommendations for improving effectiveness.

**Methods:**

Qualitative interviews were conducted with investigators from twelve multidisciplinary teams awarded pilot research funding by the University of Kentucky College of Medicine to better understand the barriers and facilitators to effective team science within an academic medical center. An experienced qualitative researcher facilitated one-on-one interviews, which lasted about one hour. Structured consensus coding and thematic analysis were conducted.

**Results:**

The sample was balanced by gender, career stage (five were assistant professor at the time of the award, seven were senior faculty), and training (six were PhDs; six were MD physicians). Key themes at the team-level centered on the tension between clinical commitments and research pursuits and the limitations for effective team functioning. Access to tangible support from home departments and key university centers was identified as a critical organizational facilitator of successful project completion. Organizational barriers centered on operationalizing protected time for physicians, gaps in effective mentoring, and limitations in operational support.

**Conclusions:**

Prioritizing tailored mentoring and career development support for early career faculty, and particularly physician faculty, emerged as a key recommendation for improving team science in academic medical centers. The findings contribute to establishing best practices and policies for team science in academic medical centers.

## Introduction

Team science is a collaborative and multidisciplinary approach to scientific inquiry that brings together researchers from diverse groups to work toward solutions to a scientific problem [1]. Transdisciplinary team science facilitates timely convergence of efforts and perspectives from different researchers to address complex problems [1,2] through an early and intentional synthesis of concepts, theories, and methodological approaches to enable scientific discovery and translation of research findings into clinical care [3]. Integrated teams of collaborative scientific experts with complementary skills in different fields working synergistically to target health outcomes are foundational to optimizing clinical and translational research [1].

Studying the process of team science facilitates an understanding of what teams require to be effective [4]. Prior research has noted that multidisciplinary teams are situated within multiple levels of influence (e.g., individual, team, department, institution, and sector) [2,5]; as such, understanding their optimal operation requires examining layered domains of interpersonal factors (e.g., interaction and communication among team members) [4], the context of participating organizations [2], and institutional influences on team science [6]. Evaluation research on team science can improve the design and effectiveness of ongoing and future collaborative research and training programs [7] and contribute to establishing best practices for team science, as well as innovative organizational policies that foster team science [5].

The current evaluation was undertaken to examine investigators’ experiences of participation in transdisciplinary team science within an academic medical center environment, including challenges, facilitators, and notable impacts, with a special focus on physicians who are often uniquely qualified to combine perspectives from clinical experience with scientific inquiry, yet are challenged by competing career priorities, pressures of clinical duties, achieving both clinical and research competence and protracted timeframes to achieve sustained research contributions [8,9]. Prior research has noted that effective organizational support for team science includes facilitating the availability of effective mentorship, fostering community through development of collaborations that cross departments, and placing value on the role of physicians by offering protected time [9]. Evaluating the impact of programs to support team science involving physicians may help institutions tailor efforts to effectively support, sustain, and grow the clinical and translational science workforce.

## Methods

### Multidisciplinary Team Science Pilot Awards

The University of Kentucky College of Medicine (COM) and the Center for Clinical and Translational Science (CCTS) undertook several recent initiatives to support innovative, collaborative research by multidisciplinary teams. In this paper, “multidisciplinary” refers to the existence of multiple distinct disciplinary backgrounds among team members, and “transdisciplinary” refers to the integration of ideas and approaches from multiple disciplines to create insights that transcend distinct fields [10]. The Multidisciplinary Value Program (MVP) was launched in 2016 to foster investigator-initiated clinical studies with specific requirements. Teams needed to include at least one member with strongly funded science, a physician investigator, and a feasible clinical/translational study proposal with relevance to the health challenges faced by Kentucky. A clear plan for future extramural grant submissions was also emphasized, recognizing the importance of funding research time for clinical members of the team. In January 2017, the Value of Innovation to Implementation Program (VI^2^P) was initiated in parallel to support collaborative team science projects between basic scientists, physicians, and implementation science experts that would identify, develop, test, and evaluate strategies to disseminate and implement evidence-based practices into public health, clinical practice, and community settings [11]. For both mechanisms, pilot awards were disease agnostic. Depending on the research focus, physicians were eligible to serve as the lead investigator, co-investigator, or in some cases, also served as the team representative with strongly funded science. Individual project awards up to $110 000 in total direct costs over an 18-month period were made on a competitive basis following an NIH-type study section review of proposals. Funds could be used for research staff salary support. Salary support for faculty was generally not allowable; however, to promote physician involvement in the projects, support for small percent effort could be requested for physician investigators who did not have protected research time. Eight MVP and 4 VI^2^P applications were funded between 2016 and 2017, and all projects were concluded by December 2019.

### Study Procedures

The CCTS Evaluation Core conducted in-depth interviews with one member of each of the 12 awarded teams to better understand the formation and strategies of the multidisciplinary scientific teams, to identify the multilevel barriers and facilitators to effective team science within an academic medical center, and to examine career enabling impacts of the awards. These interviews were part of a larger mixed methods study involving standardized surveys with all awarded team members to examine team processes, perceptions of team effectiveness and satisfaction with team collaboration, as well as scholarship outcomes of the pilot awards. A full description of the survey methods is available here [12]. This paper focuses on the qualitative study results. In-depth interviews were conducted by Zoom between September and December 2020. All 12 interviewees were members of awarded teams (one member per team), serving as either the PI, MPI, or Co-I on their respective pilot awards.

An experienced qualitative researcher facilitated the one-on-one Zoom interviews, which lasted between forty and sixty minutes. Institutional Review Board approval for the study was obtained from the University of Kentucky Medical IRB. Participants were shown the consent form on screen and the interviewer discussed the provisions of the consent document prior to beginning the interview. Participants were asked to verbally consent that they agreed to participate in the recorded interview. These semi-structured, in-depth interviews were organized by an interview guide containing broad questions on key topical areas, including team formation and composition, team process and strategies for working as a team, organizational barriers and supports for team science, recommendations for improving team science, as well as impacts of the pilot awards.

### Data Analysis

Analysis of the textual data involved several steps, including 1) verbatim transcription and quality control review of interview transcripts; 2) focused readings of these transcripts; 3) the construction and application of a detailed coding scheme; and 4) the construction of an interpretive summary based on the interview codes. Interviews were recorded and transcribed verbatim using a transcription service. Interview transcripts were reviewed and verified for accuracy by a member of the evaluation team. Following initial reading of the transcripts, the evaluation team developed a coding scheme for the interview data in NVivo [13]. To ensure robust coding, each interview was independently coded by at least two members of the team, and coding consensus meetings were held to discuss and resolve discrepancies. Following the completion of systematic coding efforts, the coded textual passages and the full narratives were read consecutively and were synthesized to identify the primary themes in the data using the principles of thematic analysis [14]. We report the primary themes in each of our topical areas (team formation and process, organizational support, and barriers for team science at the institution, recommendations for improving team science, pilot impacts).

## Results

The in-depth interview sample was balanced by gender (six female, six male), career stage (five were early career at the time of the award, seven were senior investigators), and training (six were PhDs; six were MDs). Four individuals mentioned being new faculty members at the institution at the time of award. Pilot awards were agnostic to domain area and ranged across the translational spectrum from preclinical animal studies to community-level dissemination of tested interventions.

### Team Formation and Process

In most cases, the MVP/VI^2^P teams largely consisted of a core of established collaborators, with new members added for specific disciplinary expertise when gaps were identified or in response to evolving scientific questions. All were by design multidisciplinary teams, and typically between four and five disciplinary areas were represented on the awarded teams. Two teams experienced the loss of the study PI during the project, and this instability in team leadership was mentioned as a challenge, though in both cases the teams overcame this hurdle and successfully completed the projects. In one case, the departure of the original PI allowed an early career faculty member the opportunity to assume the PI-ship and gain critical experience leading a multidisciplinary team.

By far, the most prevalent theme surrounding team formation and process was the *tension between clinical commitments and research pursuits by the physicians*. Ten of the twelve interviewees commented on the very practical challenges of time commitments for physicians, either as study leads or as engaged investigators. Physicians were seen as critical team members, and the importance of engaging physicians in research was shared repeatedly, yet practical barriers to this collaborative approach were encountered routinely by teams. As one senior faculty member observed:

*Barriers to communication I think most often come from the clinical side when you have active providers that are very committed to the project, but due to clinical responsibilities may have additional challenges. Occasionally, sometimes those clinical responsibilities are, can be planned around other times they can't. And so, I think having a backup plan with sometimes those clinical partners, if we can't meet at this time, do you have somebody that can meet and provide you that information?*

One early career physician-scientist expressly captured the internal tension she experienced on her pilot award, reflecting on the demands of balancing dual roles:

*The weeks that I had clinical service basically meant that I, there would be seven or 14 days in a row of being on inpatient service, which are very long clinical days. And it’s next to impossible to accomplish anything else besides taking care of patients. It was very intensive in terms of my actual time, which in retrospect, there’s no way now that I would be able to do that again. There are just not enough hours in a day with other responsibilities that have come up, but I also think that it’s probably true that had I not had this opportunity with the pilot study, that some of those research collaborations wouldn't have been strengthened, and then maybe some of the current activities that I'm doing wouldn't have happened.*

Four teams mentioned specific *barriers engaging clinical colleagues from other departments* as team members, noting that their studies were viewed as lower priorities, or generating concerns about possible interference with clinical care:

*I had initial commitment and participation in meetings from [other departments] and then there was really nothing, I mean, no ongoing participation from those groups. It was very difficult to get the collaborators to respond to email or participate in any meetings.*

### Organizational Supports

Turning to the question of organizational-level supports for team science, a primary theme mentioned by seven investigators was the ***orientation and support of their home department and key university centers as critical factors*** in successfully implementing and completing these pilot awards, through the provision of infrastructure and support services.

*My department’s very supportive of my research time and was supportive of me being able to apply for the MVP grant, even though it couldn't fund my effort. I think from an institutional standpoint, having these awards that are just well-respected within the university. So being able to go in, when I was talking to folks about recruitment saying, this is the MVP award, funded by the College of Medicine, supported by the Dean of our College was a helpful kind of bridge, especially as I was making new relationships. I do think offering these things, it really did help me launch my research here.*

*I would not have been able to do the pilot study without the support of [de-identified] and running clinical trials. The mentorship and day to day support setting up good clinical practices, study management, statistical support.*

At the institutional level, investigators broadly observed that the ***supportive internal funding environment*** as well as ***changing policies on team science for promotion and tenure*** were impactful as key supports for multidisciplinary team science.

*In terms of the CCTS, College of Medicine and Vice President for Research’s office, they, I think have intentionally made really great steps forward and bringing teams together. I think a lot of these pilot project programs, especially partnerships through the CCTS with different colleges has been extremely meaningful and has made an impact in getting people working together… So overall I am very encouraged by UK’s commitment and their drive to bring these teams together to collaborate.*

*The newer policies on promotion and tenure broadly help just in that it makes it easier for folks to come together, knowing that they're likely to receive some kind of credit, even if they're not the first or the senior author… as we're bringing the group together, I do think that that institutional policy has facilitated collaborative work.*

### Organizational Barriers

Beyond the specific impacts noted above to pilot team formation and function, ***protected time for physicians*** emerged as a primary organizational-level barrier to effectively supporting team science in a sustainable manner. This was reported by six interviewees, both physicians and basic science faculty alike, while executing the pilot awards, but was also described broadly as a barrier to tenable team science in the longer term:

*We don't have the time to go to NIH seminars and all this other stuff, we don't have protected time. We're not given protected time to do that. And there is no such thing for a clinician. I mean, there just isn't. People ask me, when do you write your grants? And I say one in the morning. Cause it’s the only time we have. And that’s okay to some degree, but it really isn't okay.*

*Protected time for a physician scientist is a major problem. For example, [de-identified] was on my NIH project with 15% of his time covered by the grant. That never was able to completely materialize. It’s just the way it’s set up… patient care is not four days of the week.*

Four investigators tied the issue with protected time to the tension in clinical departments around the ***dual missions of clinical care and research*** in an academic medical center environment. In many ways, the culture of clinical departments represents an ongoing challenge for early career physicians to thrive as researchers:

*If you have clinical departments that have had a long history of being a clinician-led, clinician-focused, and only clinical endeavors being a part of this academic department, it can be an uphill battle and a struggle for clinician scientists to find connection and to find resources within their department. That was certainly what I experienced.*

*Another example of trying to fit external funding into clinical programs. I needed to have people come in for research visits and there wasn't space in the clinic. And so, clinical visits would trump a research visit. And so, research staff would get kicked out of rooms because there just wasn't any way to… And these are all things that I just didn't know that I had to somehow, I didn't realize that there wasn't an infrastructure for that.*

A second theme emerged around ***limited operational support***, which typically was reported by early career female faculty team leads who mentioned inadequate support resources available that they could leverage:

*I'm a one woman show, mostly I'm still working on that. And like one thing that I find really challenging is I've got some projects that are really exciting that are really interested, really innovative that are running, but I have no coordinator to run them. So I have to run every aspect of every single project while then also trying to forward think and try to write grant applications. For me, the infrastructure is, is a pretty significant barrier to my research productivity as a faculty member.*

*The division leadership was there absolutely in terms of wanting to support me as a clinician researcher, but there wasn't an additional infrastructure. It was essentially asking people who already had more than full-time jobs to take on additional work. And it was just so incredibly difficult.*

A third key theme was reported by seven investigators across career stages, who commented openly about ***institutional gaps in mentoring-training*** that they had experienced or observed as a direct barrier to effectively supporting engagement in team-based research.

*Clinicians, there’s a need for the time, but also for mentorship and guidance. I mean, learning how to run research studies is not something that’s just universally taught in medical school. There isn't an infrastructure that’s clear, no one sits you down as a new investigator and says, these are all the things that you need to do. And these are all the people that can help you do this. There’s no roadmap.*

*Clinician scientists, you know, we want to be extramurally funded. I would love to be extramurally funded. I mean, the reality is I, you know, there are people who go through the traditional NIH mechanisms of getting K08 awards and then progress and do an R01. And a lot of us clinician scientists just will never go through that. And I'm finally starting to demand some help in terms of figuring out where I should land in terms of extramural funding. And so I think that the MVP award is fantastic and it really, it does provide a really great avenue for us to do some cool work. I think what’s missing though, is the career development piece.*

### Recommendations for Optimizing Support

During our interviews several investigators reflected on areas for improvement in terms of better supporting early career faculty, particularly physicians, with appropriate mentorship and training, optimizing support for multidisciplinary teams, and promoting collaboration and sustainability of collaboration in the longer term.

***Prioritizing the improvement of mentoring for early career faculty*** was evident as a theme across six faculty members at all career stages, including specific recommendations for initiatives to promote relationships with senior-level sponsors and programs directly incentivizing effective mentors. Physicians shared personal challenges they had experienced with identifying appropriate mentorship and career guidance:

*The nature of my department is that I couldn't really find mentorship of what I wanted to do in research within my department. I did make and really depended on some mentoring relationships within my department on aspects of it, but there were very key leaders within the university who became mentors for me. So I think that’s an important thing that will build team science.*

*I kind of wished there was a little bit more, I say mentorship, but it doesn't have to be that regular. It would just be like, okay, let’s look at [de-identified] or let’s look at [de-identified] or let’s look at [de-identified]. These are all people in my department, you know, what kind of research are they doing? Let’s sit them down, ask them what they want to do with research. What questions would they want to ask, how are they going to ask them, what are the grant mechanisms that they really, that would help them get funded. I mean, many of us didn't go to med school to become scientists. I think there’s gotta be some more oversight to make sure that we're getting good advice and good planning as far as what to do with our careers. I mean, that’s really what, what would help a lot.*

In the longer term, six investigators we spoke with emphasized the need for ***allocation of additional resources*** beyond the project-specific scope of the MVP/VI^2^P mechanisms to support continued research growth in clinical departments and the sustainability of new team collaborations. Interesting, too, is the recognition that support needs to be tailored, not every investigator has the same gaps or needs.

*Sustainability and building on more long-term collaborations would be a recommendation I would have. And so I think you can have an excellent infrastructure. You can have excellent leads in different areas. You can bring those leads together initially, but to make it sustainable. I think it really requires that, intentional allocation of time, which is money.*

*Are there things that UK that we could do better? At [de-identified] it was about figuring out what they wanted to do and then shaping their questions and answers in a way that made the science and think of build preliminary data. And then having a grant writer help them actually write the story that would be R01-worthy. And so it was a very heavy handed approach to get these people funding. But I think for us clinicians, that’s probably what we need.*

*My path as a physician scientist has been a little atypical. I think a lot of people expect, you know, first you get a K and then that’s the kind of traditional track, right? That didn't happen for me for a number of reasons. I think there’s a lot of, for a lot of junior people, there’s a lot of ‘you need to focus a lot on grant writing’ and that sort of thing. For me, it was much more about the operational stuff. The writing skill was not my gap, the operational, practical, how do I make this all happen?*

### Pilot Award Impact: Career Development

Despite the noted challenges, for early career PIs in particular, the MVP and VI^2^P pilot awards provided clear support for advancing their scientific agendas. All five early career interviewees noted that the ***awards were career enabling in some regard*** by affording a mechanism to launch research at the institution, providing experience leading a team, building a collaboration, and achieving recognition for their research accomplishments:

*Since the MVP, I have gotten tenured and promoted to an associate professor and I'm sure that that was helpful in my path. I think I have always through my entire career, reached out for mentorship and had multiple mentors along the way. And I think the MVP program helps to connect with mentors particularly at this institution. I do think one of the things that was unique about the MVP is I was really seeking multidisciplinary folks for the team. And so it also gave me the opportunity to also seek some multidisciplinary mentorship, which I think definitely has value as well.*

*The interactions that I gained through the MVP, the setup, if you will, of that work really has fueled my ability to be the PI for [deidentified]. I was recently approached and am going to be a national PI for another study that’s somewhat related. So I think, now that’s an industry funded trial, but that’s okay. That collaboration really started through the projects that I was working with through the MVP, but I think that’s how science grows.*

## Discussion

The present study examined investigators’ perspectives on participation in transdisciplinary team science initiatives within an academic medical center environment and systematically identified the most salient barriers and supports encountered by physician and basic science investigators during implementation of team science pilot research projects. The findings highlight unique challenges and impacts of team science initiatives drawn from the investigators’ lived experience with transdisciplinary research collaboration and clearly illuminate the critical influence of organizational resources and support in effectively promoting team science [15].

Notably, this study documented the tension between clinical commitments and research pursuits as a critical and prevalent obstacle to successful team formation and collaboration. The strain to team formation and functioning appears to be rooted in time constraints, competing organizational priorities, conflict between the role of physician and investigator, as well as lack of appropriate reward and recognition structures, which resonates with earlier research on engaging physicians in team science initiatives [1,16]. Prior research has noted the need for organizations to mitigate the tension between clinical and research priorities and to identify approaches for effectively navigating dedicated time for research within the realities of patient care and clinical duties [9].

In the present study, protected time for physicians emerged as the most prevalent organizational barrier to effective team science, and particularly hampered physicians in pursuing a robust research trajectory. Our findings resonate with prior efforts which found that effective organizational support for team science must involve valuing the dual role of physician-scientists by offering protected time [9], but the means to effectively operationalize protected time for research within systems driven by clinical metrics and revenue generation remain challenging for academic medical centers. In addition, as described above, the demands of patient care do not necessarily stop when the faculty member is not assigned to a clinic, again emphasizing the operational challenges of “protected time.” Importantly, our findings also revealed a general sense of ambivalence from physician investigators that the current models and mechanisms of career development and research support available for basic scientists were not especially appropriate pathways for physicians and are not optimal for their developmental trajectories in research.

Investigators across teams, most notably among physicians, felt that institutional gaps in mentoring were rate limiting to their effective engagement in research. To further optimize support, suggested areas of improvement included enhancing relationships with senior-level sponsors outside of their home departments, more tailored support for each investigator’s needs, and programs directly incentivizing effective mentors. Senior basic and clinical faculty expressed a strong sense of personal responsibility for contribution toward the development of more junior colleagues by taking on direct mentoring roles, citing their lived experience with similar challenges as junior faculty interested in research within heavily clinical departments.

Research networking and collaboration between early career investigators and those who are more advanced in research may provide robust career enabling support systems [6,17]. Additionally, training and mentorship may improve researchers’ knowledge and skills necessary to succeed in research [6,18]. Incentivizing mentors through provision of career development awards, providing salary support for mentoring activities, and rewarding outstanding mentors may encourage dedication from effective mentors, provide new mentoring opportunities, and facilitate an organizational culture of mentoring excellence [9,19-21]. Additionally, having multiple mentors may be beneficial for investigators in clinical departments without robust research track records [22].

A major career enabling impact of the MVP and VI^2^P as highlighted by our study was provision of support to early career PIs to advance their research agenda, to become more established at the institution, gain experience leading a multidisciplinary team, building collaboration, and achieving recognition for their research accomplishments. Our interviewees noted a need to allocate additional resources beyond the project-specific pilot awards to support sustained research growth, which resonates with prior research documenting the critical role of institutional resource commitment in enhancing collaboration, productivity, research impact, and sustainability of research teams [6,23-26]. Importantly, we noted that early career female faculty experienced specific barriers related to operational support that did not emerge among other interview participants, which may indicate a need to further examine equitable access to resources within institutional programs.

### Limitations

The present study is limited by several factors. First, the study took place in a single academic medical center in Central Kentucky providing a snapshot of the experiences of team scientists within one institution with its unique organizational context. In addition, we conducted interviews with a single member of each investigator team, which limits our ability to uncover alternative opinions and perspectives. As such, application of the findings to other academic medical settings may be limited given our relatively small sample. Second, the narrative responses are self-reported experiences of investigators, which may be influenced by social desirability, self-presentation, and recall biases to an unknown extent, and did not include perspectives of organizational leaders. However, strategies employed to enhance the accuracy of reports included assurances of confidentiality and the use of a neutral interviewer.

### Implications and Conclusion

Central to the multidisciplinary research team is the physician scientist who can effectively translate bench research to improve bedside clinical care. Notably, however, few physicians pursue careers in clinical and translational research [27], which represents a formidable challenge in developing effective translational research teams. There is little evidence to suggest that physicians are increasingly gravitating to research careers, despite widespread recognition that physician engagement in research is critical to advance clinical care [28]. Given that frontline physicians have the knowledge and lived experience of providing medical care that researchers seek to improve, it is critical to ensure their involvement in research geared toward translating healthcare innovations to improve patient care and clinical outcomes [29,30]. This study noted that the engagement of early-career physicians in multidisciplinary teams had positive impact on interest in pursuing further research and that efforts to facilitate transdisciplinary team science by involving physicians within an academic medical center through the provision of dedicated funding resources was effective in increasing physician engagement, suggesting that barrier-removing engagement from clinical, research, and organizational leadership [31] may be essential to continued progress.

Given these observations, we offer the following key considerations for enhancing effective team science in an academic medical center environment: 1) obtain departmental commitment to research effort for physician scientists, at least for a defined period of time; 2) define clear targets and timeline for external funding to support and sustain protected time for physician scientists; 3) create a clear mentoring plan for early career physician team members, led by accomplished and engaged senior researchers; 4) engage in a systematic assessment of needs for individual and team operational support; and 5) define and examine key metrics of the overall pilot program success over at least a two-year period, including numbers of grant submissions, numbers of NIH awards, percent of protected time covered by external grant funding, number of co-authored publications, and number of early career faculty identified and mentored.

Implementation of these recommendations will require substantial institutional innovation and commitment. Although some skills, such as understanding NIH guidelines, grant writing, and time management, can be taught through robust training offerings, early-stage investigators are best served by working closely with their mentor(s). Hence, there is a clear need to develop robust matrix mentorship models to aid in career enhancement for physician scientists. Doing so will require making commitments to support mentors, by considering mentoring activities in promotion decisions and incentivizing quality mentorship through awards and salary support. It has been noted that successful mentorship in academic medical centers requires elevating mentorship as a strategic priority, around which the development of appropriate institution-specific goals, objectives, and metrics can occur [32,33]. In a similar way, innovations to achieve authentic protected time must be elevated as institutional priorities in order to achieve success, moving away from ad hoc or informal agreements toward the development of formalized internal guidelines that are institutionally endorsed. Notably, this commitment will necessitate better coordination of funding streams and support mechanisms to defray significant costs that institutions bear during the physician scientist training and incubation period [34].
